# A comprehensive review on motion trajectory reconstruction for EEG-based brain-computer interface

**DOI:** 10.3389/fnins.2023.1086472

**Published:** 2023-06-02

**Authors:** Pengpai Wang, Xuhao Cao, Yueying Zhou, Peiliang Gong, Muhammad Yousefnezhad, Wei Shao, Daoqiang Zhang

**Affiliations:** Key Laboratory of Brain-Machine Intelligence Technology, Ministry of Education, College of Computer Science and Technology, Nanjing University of Aeronautics and Astronautics, MIIT Key Laboratory of Pattern Analysis and Machine Intelligence, Nanjing, China

**Keywords:** brain-computer interface, EEG, trajectory reconstruction, motion execution, motion imagery

## Abstract

The advance in neuroscience and computer technology over the past decades have made brain-computer interface (BCI) a most promising area of neurorehabilitation and neurophysiology research. Limb motion decoding has gradually become a hot topic in the field of BCI. Decoding neural activity related to limb movement trajectory is considered to be of great help to the development of assistive and rehabilitation strategies for motor-impaired users. Although a variety of decoding methods have been proposed for limb trajectory reconstruction, there does not yet exist a review that covers the performance evaluation of these decoding methods. To alleviate this vacancy, in this paper, we evaluate EEG-based limb trajectory decoding methods regarding their advantages and disadvantages from a variety of perspectives. Specifically, we first introduce the differences in motor execution and motor imagery in limb trajectory reconstruction with different spaces (2D and 3D). Then, we discuss the limb motion trajectory reconstruction methods including experiment paradigm, EEG pre-processing, feature extraction and selection, decoding methods, and result evaluation. Finally, we expound on the open problem and future outlooks.

## 1. Introduction

For a long time, scholars in the fields of neuroscience and computer science have been exploring how to understand the brain and uncover the neural information within the brain (Perry et al., 2010). The development of computer technology makes research in the field of neurology increase year by year. With the help of powerful computers and machine learning, researchers can interpret and use signals extracted from the brain, making it possible to create brain-controlled devices and enable disease rehabilitation.

Brain-computer interface (BCI) is a communication system that uses different brain signals to convey human intentions to computers or machines (Korik et al., 2014; Tariq et al., 2018; Chen et al., 2019; Chaudhary et al., 2021; Xu et al., 2022). The BCI system collects brain activity signals through implanted electrodes or external devices. It then converts these signals into computer-controlled commands in real-time, allowing information to be transmitted directly through the brain instead of peripheral nerves and muscles (Cho et al., 2018; Meng and He, 2019; Nagel and Spüler, 2019; Garcia-Moreno et al., 2020; Zhang, 2021). This technology has been widely explored in the past few decades. The BCI system has great potential for applications in many fields, including clinical rehabilitation training programs (Bai et al., 2020; Mane et al., 2020; Brusini et al., 2021), typing communication systems (Wolpaw et al., 2002; Milekovic et al., 2018; Zhang et al., 2018; Renton et al., 2019), robotics (Bi et al., 2013; Wang et al., 2018; Chamola et al., 2020; Baniqued et al., 2021; Robinson et al., 2021), entertainment (Noor et al., 2018; Pradhapan et al., 2018; Wang et al., 2019; Li et al., 2021), and so on. The recording methods of brain activity can be divided into two main categories: invasive and non-invasive (Zhuang et al., 2020). Electroencephalography (EEG) and electrocorticography (ECoG) are the most common non-invasive and invasive recording methods, respectively (Nicolas-Alonso and Gomez-Gil, 2012). However, due to surgical risk and the gradual degradation of signal quality over time, invasive methods have significant shortcomings (Abiri et al., 2019b). EEG can measure neural activity directly with high-time resolution and can be operated in real-time while non-invasive, cheap, and portable. It has been shown to be the most popular method (Abiri et al., 2019a).

Studies have shown (Bandara et al., 2018; Abiri et al., 2019b; Sandhaeger et al., 2019) that EEG signals carry a variety of motion information, including position, velocity, acceleration, angular velocity, etc., which provides theoretical support for controlling external machinery such as prosthetics through EEG-based BCI. EEG-based BCI can provide access from the brain to external devices, providing brain-controlled aids for patients with dyskinesia (due to stroke, neurological disease, or brain trauma). Among many BCI paradigms, sensorimotor rhythm (SMR) BCI based on multi-class classification is widely used in the robot control domain. This method mainly uses the power density of mu (8–12 Hz) and beta (18–26 Hz) EEG bands in the central and parietal cortex (Xu et al., 2021). Subjects need to learn to adjust these bands independently, and may take weeks or even months. Moreover, this method is only suitable for discrete control of external devices, typical applications are wheelchairs and mice, which can not effectively control artificial arms. In practice, for some complex and high-precision activities, we hope to control the device to move smoothly and continuously. This requires richer motion information, therefore motion trajectory prediction (MTP) BCI is the ideal solution. MTP-BCI can predict the current motion state, such as position, speed, acceleration, and more, from the EEG characteristics of the last several time lags, to achieve the continuous reconstruction of the imagined or executed motion trajectory. So far, researchers have explored and published many related publications in this field, which can be categorized into the hand (Bradberry et al., 2010; Lv et al., 2010; Yuan et al., 2010; Heger et al., 2012; Ofner and Müller-Putz, 2012, 2014; Kim et al., 2014a,b; Robinson et al., 2013, 2014, 2015, 2021; Korik et al., 2015, 2016, 2018; Sun et al., 2017; Úbeda et al., 2017; Mondini et al., 2020; Sosnik and Zur, 2020; Sosnik and Zheng, 2021), the arm (Ofner and Müller-Putz, 2012, 2014; Kim et al., 2014b), the shoulder (Mondini et al., 2020; Sosnik and Zur, 2020; Sosnik and Zheng, 2021), the elbow (Mondini et al., 2020; Sosnik and Zur, 2020; Sosnik and Zheng, 2021), the finger (Paek et al., 2014), the reconstruction of the trajectory and velocity of the lower limb (Presacco et al., 2011; Castermans et al., 2014), including the motion in the 2D plane and 3D space, and the on-line control of the continuous motion of the manipulator (Mondini et al., 2020).

Although there have been a lot of pioneering work in this field in the past decade, as far as we know, there is not an overview of the methodology of trajectory reconstruction. For example, as described in Korik et al. (2014), the design of the decoder and the decoding accuracy obtained by some studies are briefly introduced. To alleviate this gap, this paper investigates the literature on MTP-BCI, covering the 2D and 3D trajectories generated by motion imagination and motion execution, in order to summarizing a set of trajectory reconstruction processes as detailed as possible, and report the main research progress in this field.

**Figure 1:**
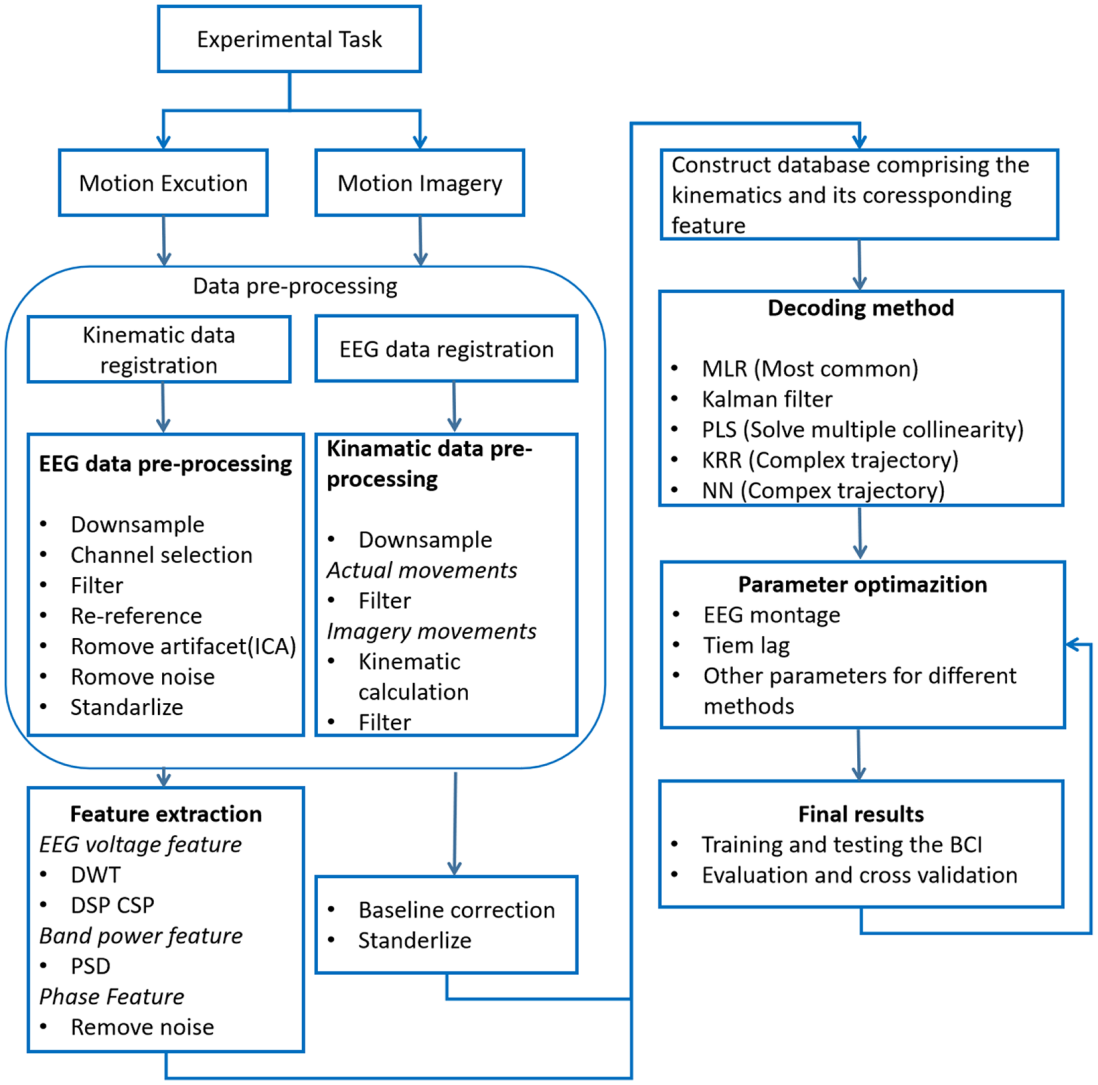
Schematic diagram of EEG signal-based Trajectory reconstruction system.

In Figure 1, the basic process of trajectory reconstruction is illustrated. The experimental task can be divided into motion execution (ME) and motion imagination (MI). During the execution of the task, neural signals (EEG signals) and dynamic data (motion trajectories) are recorded in parallel. Then, the data is pre-processed, and the appropriate feature extraction method is adopted for EEG signal. The common features are EEG potential and band power. Next, we need to choose the appropriate decoding method, that is the most important part to determine the reconstruction accuracy. The decoder is then trained offline to achieve maximum correlation between the measured trajectory and the reconstructed trajectory. Finally, we reconstruct the motion trajectories online through the whole process, and dynamically optimize the parameters according to the results.

The rest of this review is arranged as follows. In the second part, we summarize the main methods used in each process of 2D trajectory reconstruction. Then, we supplement other methods used in 3D trajectory reconstruction in the third part. In the fourth part, we summarize the main findings of this review and discuss open issues that require further investigation. Finally, we summarize the whole thesis in the sixth part.

## 2. 2D limb motion trajectory reconstruction

**Figure 2:**
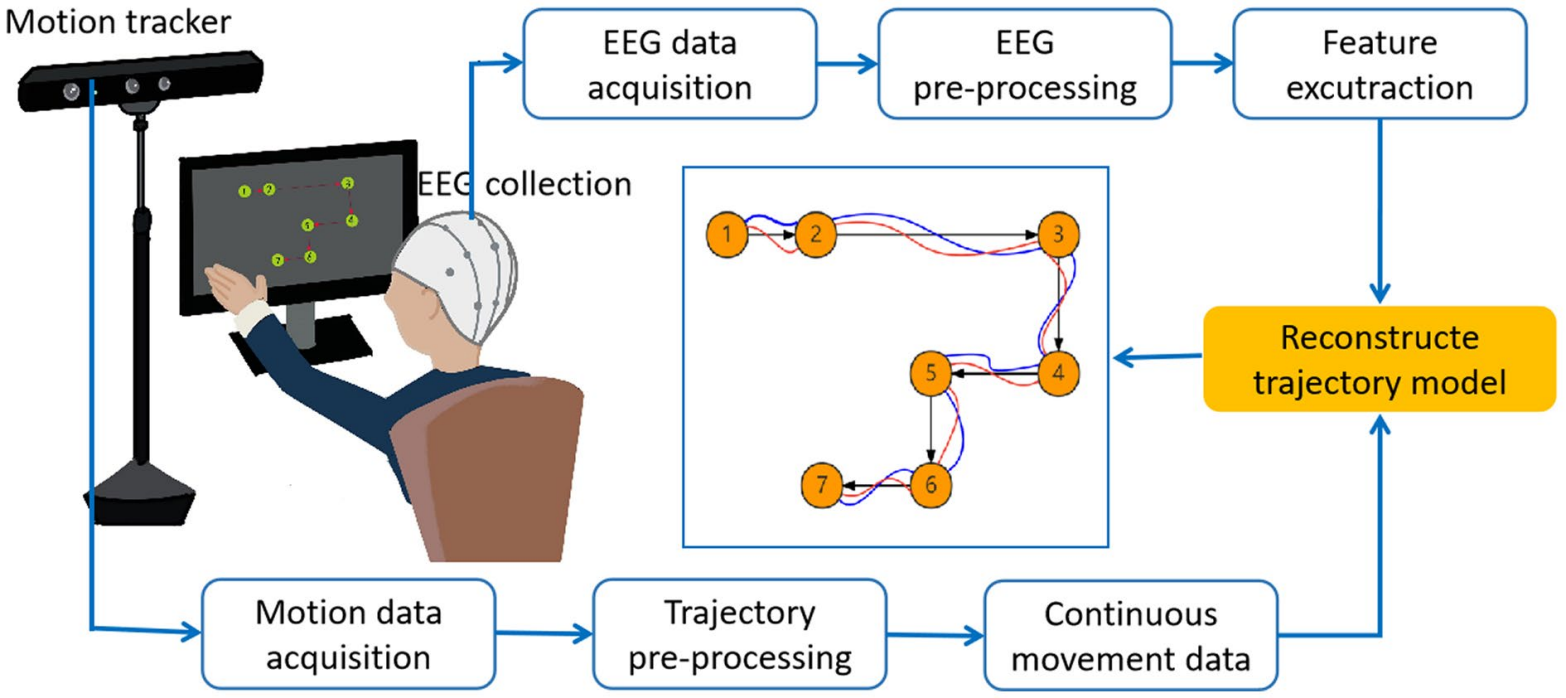
A common system for hand movement decoding from EEG and motion trajectory. Movement directions are displayed as black arrows. The movement trajectories performed by a subject are displayed as blue lines. The reconstructed trajectories are displayed as red lines.

In recent years, many articles have been published in the field of limb trajectory reconstruction. Therefore, a variety of experimental paradigms have been designed for various limbs, such as hands, shoulders, elbows, fingers, ankles, knee joints, and hip joints. We divide them into two categories: 2D limb trajectory reconstruction and 3D limb trajectory reconstruction. A common system for hand movement decoding from EEG and task paradigm are shown in Figure 2.The process of trajectory reconstruction from EEG signal includes signal pre-processing, feature extraction, and signal decoding. In this part, we will introduce in detail the experimental paradigm, signal pre-processing, feature extraction, and signal decoding methods in 2D limb trajectory reconstruction.

### 2.1. Experimental paradigm and decoding performances

We summarize the research on 2D plane trajectory reconstruction from seven aspects: task type, limb, experimental paradigm, number of subjects, decoding methods, EEG features, and decoding performance, as shown in Table 1.

**Table 1:** The studies for decoding limb movement trajectory on 2D tasks from EEG.

Authors	Tasks	Limb	Methods	Subjects	Features	Experimental paradigms	Decoding performances
Lv et al. (2010)	ME^①^	Hand	KF^②^, Smoother	5	DSP^③^, CSP^④^	Move a pen at their own pace along a zigzag route.	Pearson’s r: x:0.37 ± 0.08 y:0.24 ± 0.06 SNR^⑤^: x:0.81 ± 0.38 y:0.27 ± 0.13
Robinson et al. (2013)	ME	Hand	MLR^⑥^	7	DWT^⑦^	Centre out right hand movements in horizontal 2D space.	Pearson’s r:0.56 ± 0.16
Robinson et al. (2015)	ME	Hand	KF	7	DWT	Center-out right-hand movement tasks in four different directions at two different speeds in random order.	Pearson’s r: 0.60 ± 0.07
Robinson Neethu et al. (2014)	ME	Hand	MLR	7	DWT	Center-out right hand movements in horizontal 2D space.	Pearson’s r: 0.63
Sun et al. (2017)	ME	Hand	MLR	5	Phase	Control the position of a cursor moving towards four different directions according to the target-cue on the screen.	Pearson’s r: (position) x: 0.46 ± 0.11 y: 0.43 ± 0.08 velocity: x: 0.48 ± 0.11 y: 0.44 ± 0.09
Robinson et al. (2021)	ME	Hand	Linear SVM^⑧^, KF, MLR	21	BP^⑨^, FBCSP^⑽^	Follow the line and move your hand to touch the target in the GUI.	Acc^⑾^: F-S^⒅^: 73.36% FR-SR^⒆^:69.46% FL-SL^⒇^:68.99% Pearson’s r: KF:0.3799 ± 0.08 (best case) MLR:0.3968 ± 0.08 (best case)
Mondini et al. (2020)	ME	Hand	PLSKF^⑿^	10	EV^⒀^	Track a moving object with a robotic arm through EEG-based decoded trajectories.	Pearson’s r:0.32
Paek et al. (2014)	ME	Finger	LD^⒁^	5	ED^⒂^	Tap right index finger three times in succession.	Pearson’s r: 0.36
Ofner and Müller-Putz (2014)	MI^⒃^	Arm	PLS^⒄^	9	EV	Imagine horizontal or vertical repetitive rhythmic arm movements.	Acc:64% ± 10%
Úbeda et al. (2017)	ME	Upper limb	MLR	5	EV	Subject actively or passively grasped the planner to perform a center-out task.	Acc: Configuration A:29.0% ± 11.8%(chance level 12.5%) B: 51.3% ± 19.2% (chance level 25%) C: 52.3% ± 20.5% (chance level 25%) D: 79.6% ± 15.9% (chance level 50%) E: 75.6% ± 17.0% (chance level 50%)

①Movement execution, ②Kalman filter, ③Discriminative spatial pattern, ④Commonspatial pattern, ⑤Signal-noise ratio, ⑥Multiple linear regression, ⑦Discrete wavelet transform, ⑧Support vector machines, ⑨Band power, ⑩Filter-bank common spatial pattern, ⑾Accuracy, ⑿Partial least squares, ⒀EEG voltage, ⒁Linear decoder, ⒂EEG derivative, ⒃Motor imagery, ⒄Partial least squares, ⒅Fast-slow, ⒆Fast right-Slow right, ⒇Fast left-slow left.

### 2.2. Signal pre-processing

During the 2D motion task, the recorded EEG signal is contaminated by various artifacts such as Electrooculogram (EOG) and Electromyography (EMG), which may confuse the EEG decoding of the trajectory (Lv et al., 2010). Therefore, it is necessary to pre-process the EEG signal before reconstructing the motion trajectory in order to obtain a higher Signal Noise Ratio (SNR). So far, the main pre-processing methods are filtering and Independent Component Correlation Algorithm (ICA).

#### 2.2.1. ICA

ICA is a method to transform multivariate random signals into a linear combination of statistically independent non-Gaussian signal sources. Using this method, independent components can be extracted from mixed signals (Subasi and Ismail Gursoy, 2010). ICA can be used to remove artifacts, such as EOG and EMG to improve signal quality and improve the correlation of trajectory reconstruction. Many works about trajectory reconstruction have adopted ICA as the signal pre-processing algorithm, such as Lv et al. (2010), Ofner and Müller-Putz (2014), and Paek et al. (2014). There are two steps in the use of ICA, first decomposing the EEG signal into several independent components, and then manually checking and removing artifacts. Many tools for removing artifacts are provided in Matlab’s EEGLAB toolkit (Nicolas-Alonso and Gomez-Gil, 2012) and Python’s MNE toolkit (Delorme and Makeig, 2004).

#### 2.2.2. Filtering

Due to the existence of power frequency interference (50 Hz in Asia and Europe, 60 Hz in the United States) and high-frequency noise, filtering has become the most common signal pre-processing method in trajectory reconstruction tasks, including EEG signal filtering and motion trajectory filtering.

Band-pass filtering and notch filtering are the most commonly used EEG filtering methods. Studies have shown that the neural correlation of kinematics mainly exists in SCP above 0.1 Hz (Garipelli et al., 2013). Therefore, most work adopts band-pass filtering to retain the correlation part of the EEG signal while removing high-frequency noise. For example, in Mondini et al. (2020), EEG goes through two-stage filtering (0.18 Hz high-pass + anti-aliasing low-pass filter and 1.5 Hz low-pass filter), and [0.5–20] Hz band-pass filtering is carried out in Robinson et al. (2021). In Úbeda et al. (2017), The EEG signal is filtered by band-pass at 0.1–2 Hz. Besides, it is also filtered between 8–12 Hz, 14–30 Hz and 0.1–40 Hz to estimate the amount of information present in each frequency band, and so on (Ofner and Müller-Putz, 2014; Paek et al., 2014; Sun et al., 2017). Notch filter (50/60 Hz) is usually used to attenuate power line noise (Robinson et al., 2015), but it is easy to cause waveform distortion (Ai et al., 2018). Low-pass filtering is usually used for kinematic data. In Mondini et al. (2020), low-pass filtering is performed at 4 Hz for motion tracks recorded using Leap Motion. In Paek et al. (2014), kinematic data are filtered at 3 Hz (that is, in the delta band), because subjects in this frequency band retain more than 95% of the cumulative power in the finger movement PSD, and can reasonably maintain the integrity of the kinematic track during visual inspection. In Úbeda et al. (2017), cursor kinematics (position and velocity) also uses a zero-phase fourth-order Butter-worth filter lower than 2 Hz for low-pass filtering.

### 2.3. Feature extraction

After data pre-processing, we obtain data with a higher signal-to-noise ratio (SNR). Next, we need to extract the salient features from the EEG signal to construct the predictor group (predictor set). At present, most of the features are concentrated in the frequency domain. We will introduce the feature extraction methods in detail below.

#### 2.3.1. DWT

EEG signal is non-stationary, which brings difficulties to signal analysis because we need to extract stationary features from the signal constantly. For the trajectory reconstruction task, most of the work is focused on the reconstruction of trajectory motion parameters from low-frequency EEG signals, so it is particularly important to improve the resolution of low-frequency signals (Robinson et al., 2013).

DWT (Discrete Wavelet Transform) decomposes the original signal into a set of prototype functions through continuous high-pass and low-pass filtering, which is called wavelet function cluster. Wavelet can represent the local characteristics of the signal in the time domain and frequency domain, and the trade-off of time-frequency resolution can be realized by selecting the appropriate scale, so as to solve the problem of instability of EEG signal. After that, the signals of different sub-bands are obtained by inverse transform reconstruction (Robinson et al., 2015). Robinson et al. (2013, 2014, 2015) used the orthogonal wavelet cluster to filter the EEG signal in the non-overlapping subspace, and the sub-band is defined as a predictor set, which achieves better decoding performance than other methods at that time.

#### 2.3.2. DSP and CSP

Discriminative Spatial Pattern (DSP) and Common Spatial Pattern (CSP) are two linear projection methods with different purposes. DSP projects the EEG signal to the linear subspace to maximize the inter-class variance and minimize the intra-class variance to extract the amplitude of slow non-oscillatory sources. CSP uses the diagonalization of the matrix to find a set of optimal spatial filters for projection, so that the variance value difference of the two types of signals is maximized, thereby obtaining a feature with a high degree of discrimination vector. Let *X∈R^C × N^* represent the matrix of collected EEG signals, where the channel number is *C* and the number of samples is *N*. The classic CSP problem is formulated as follows:

(1) $$\underset{\omega \in R^{C}}{\max}=\frac{\omega^{T}m_{1}\omega }{\omega^{T}m_{2}\omega }$$

where ω is the spatial filter coefficient and *M_i_* (*i* = 1, 2) represents the one-class covariance matrix. In general, generalized eigenvalue decomposition (EVD) can solve this problem.

(2) $$M_{1}\omega =\lambda \left(M_{1}+M_{2}\right)\;\omega$$

where λ is an eigenvalue of $M_{1}$ and $M_{2}$ . The C eigenvector is a generalization obtained by solving Equation 2.

Lv et al. (2010) filter the ICs retained after ICA into 10 frequency bands (0.1–4 Hz, 4–8 Hz, 8–12 Hz, …36–40 Hz), then extract slow assignment features in 0.1–4 Hz band by DSP, and uses CSP to extract oscillation power features in other frequency bands of IC. The FBCSP algorithm is used in Bradberry et al. (2010). The selected frequency band is [2b, 2b + 2] Hz, b = [0, 8], and the CSP parameter is selected as 3 to extract features.

#### 2.3.3. Phase feature

Most previous studies have selected the amplitude characteristics of EEG signals when selecting features (Ofner and Müller-Putz, 2014; Paek et al., 2014; Úbeda et al., 2017; Mondini et al., 2020). However, the amplitude feature only represents the intensity of neural activity, and the phase information has not been widely applied in this field of research. Previously, Sburlea et al. (2016) have proved that the phase feature has a higher SNR than the amplitude feature in the discrete gait intention detection task. Sun et al. (2017) extracted the hand motion parameters of instantaneous phase feature decoding by Hilbert transform, and obtained higher decoding accuracy than amplitude features.

### 2.4. Decoding methods

Reconstructing the limb motion trajectory with high correlation through EEG signal is the main task of exploiting high-performance trajectory reconstruction BCI. After two steps of pre-processing and feature extraction, we get the feature set, then we need to design an effective decoding model. Since we need to get continuous predicted trajectories, this is a regression problem. In this section, we introduce some commonly used motion trajectory reconstruction algorithms in detail, such as Kalman filter, Multivariate Linear Regression (MLR), and Partial Least Squares (PLS).

#### 2.4.1. Kalman filter

Kalman filter is an estimation algorithm using the linear system, which optimally estimates the current state of the system through the system input and the last prediction result. This is a recursive process, and the filter model is continuously optimized by new observations. The Kalman filter consists of two parts, including the process equation and the measurement equation, which describe the evolution of the internal state over time and describe the relationship between the noise measure and the state. The Kalman filter equation can be written in discrete time and linear form as:

(3) $$\{\begin{array}{c} \theta_{k+1}=F\theta_{k}+v_{k}\;v∼N\left(0,Q\right)\\ z_{k}=H\theta_{k}+w_{k}\;w∼N\left(0,R\right) \end{array}$$

where *θ* denotes the *n_θ_*-dimensional state of the system, F is the transition matrix between the current *k* and the next *k + 1* time samples, and v is the additive Gaussian noise modeling uncertainty and error propagation. *z* is an *n*_z_-dimensional vector of measured values, *H* is a matrix that simulates the linear relationship between *z* and *θ*, and w is the additive Gaussian noise of simulated measured values and model errors.

In the trajectory reconstruction study, the Kalman filter was used to estimate the motion parameter, ie, the state *θ*, from the noise measurement *z*, ie, the multi-lag EEG. The Kalman filter has previously been applied to decode hand movements in invasive BCI (Wu et al., 2002, 2003). The Kalman filter models discrete-time linear systems, which assume that the measured output of the system (EEG signal) is linearly related to the state (motion trajectory). Many BCI works based on trajectory reconstruction (Lv et al., 2010; Robinson et al., 2015; Mondini et al., 2020) have employed the Kalman filter.

In Lv et al. (2010), Lv et al. choose the decoding method of the Kalman filter and smoother and used the smoothing method to integrate the past, present, and future information of hand speed into the Kalman model, obtaining a better correlation and SNR than the linear filter and the Kalman filter. The decoding performance of different frequency bands is also compared in this paper. It is found that in addition to the 0.1–4 Hz band, the oscillation rhythm of the 24–28 Hz band also carries hand speed information (Lv et al., 2010).

In the method proposed by Robinson et al. (2015), the Kalman filter is used to adaptively estimate hand motion parameters from EEG signals. Too many prediction variables will provide redundant and non-stationary information, which will affect the performance of the Kalman filter and deteriorate it. Given this, Robinson et al. (2015) proposed to select the channel *a priori* through the sorting algorithm, and then eliminate the prediction variables backward to select the prediction variables with the largest amount of information to model the estimator, which significantly reduces the number of predictors and estimation time under the condition that the prediction accuracy is unchanged.

#### 2.4.2. Multivariate linear regression

The purpose of the MLR model is to construct a regression equation and use multiple independent variables to estimate dependent variables to explain and predict the value of dependent variables. MLR can be described by the following formula:

(4) $$x\left[t\right]-x\left[t-1\right]=a_{x}+\overset{C}{\underset{c=1}{\sum }}\overset{L}{\underset{k=0}{\sum }}b_{ckx}S_{c}\left[t-k\right]$$

(5) $$y\left[t\right]-y\left[t-1\right]=a_{y}+\overset{C}{\underset{c=1}{\sum }}\overset{L}{\underset{k=0}{\sum }}b_{cky}S_{c}\left[t-k\right]$$

(6) $$z\left[t\right]-z\left[t-1\right]=a_{z}+\overset{C}{\underset{c=1}{\sum }}\overset{L}{\underset{k=0}{\sum }}b_{ckz}S_{c}\left[t-k\right]$$

where *x*[*t*]-*x*[*t*-1], *y*[*t*]-*y*[*t*-1], and *z*[*t*]-*z*[*t*-1] are the position axes of time *t* in *x*, *y*, and *z*. *L* is the time lag number, *S_c_*[*t-k*] is the standardized voltage difference measured by the EEG sensor *c* at time lag *k*, and the variables *a* and *b* are the weights obtained by multiple linear regression. *C* is the number of electrodes used in the analysis.

In the trajectory reconstruction task, the dependent variables are usually the reconstruction parameters of interest, such as position, velocity, acceleration, etc., and the independent variables are usually EEG signals with different time lags from different channels or features extracted from EEG signals, such as the direct use of EEG signals (Úbeda et al., 2017), the signal amplitudes of different frequency bands obtained by DWT (Robinson et al., 2013), and the instantaneous phase features obtained by Hilbert transform (Sun et al., 2017).

With its simple and effective characteristics, multiple linear regression has always been the most commonly used decoding method in trajectory reconstruction. However, due to the large channel correlation of EEG signals, the weights of multiple linear regression are unexplained, which is called multicollinearity. In order to solve the problem of multicollinearity, in recent years, more and more work began to use other decoding methods, such as PLS.

#### 2.4.3. Partial least squares

For EEG signals, multiple collinearities have become a serious problem because of the large channel correlation, resulting in the unexplainable weight of multiple linear regression (Farrar and Glauber, 1967). PLS is particularly suitable for such situations, such as multiple lags, and low-frequency EEG (Mondini et al., 2020). PLS provides a method of many-to-many linear regression modeling. It studies the correlation between variables by using potential variables to consider the internal structure of the data. In addition, it can deal with noisy and multi-linear variables, which has advantages over traditional classical regression analysis methods.

In the study of motion imagination by Ofner and Müller-Putz (2014), the trajectory of the right arm is decoded by PLS to classify horizontal and vertical imaginative rhythmic movements. The model in Mondini et al. (2020) combines the dimension reduction characteristics of PLS regression and the data fusion characteristics of KF, and is used to decode the hand motion from EEG online, which is called PLSKF. This method integrates linear models with different motion parameters, which can significantly improve the correlation than using PLS alone, and the decoded trajectory has a more smoothing effect.

## 3. 3D limb motion trajectory reconstruction

Compared with 2D limb motion, 3D limb motion has higher degrees of freedom, so the trajectory formed by 3D limb motion is more complex, and the method used to reconstruct 3D limb motion trajectory is also more complex. In this section, we will introduce in detail the experimental paradigm and reconstruction process in the research on 3D limb trajectory reconstruction in recent years.

### 3.1. Experimental paradigm and decoding performances

We summarize the research of 3D trajectory reconstruction from the same aspect, and draw as shown in Table 2.

**Table 2:** The studies for decoding limb movement trajectory on 3D tasks from EEG.

Authors	Tasks	Limb	Features	Methods	Subjects	Experimental paradigms	Decoding performances
Presacco et al. (2011)	ME	Lower limbs (hip, knee, and ankle joints)	EV	MLR	6	Walk on a treadmill at their self-selected comfortable speed while receiving visual feedback of their lower limbs.	Pearson’s r:0.75 ± 0.1 SNR values (dB): 4.13 ± 2.03
Presacco et al. (2012)	ME	Lower limbs (hip, knee, and ankle joints)	EV	MLR	6	Walk on a treadmill at their self-selected comfortable speed while receiving visual feedback of their lower limbs.	Worst-case: Pearson’s r:0.6 SNR values (dB): 2
Bradberry et al., 2010)	ME	Hand	EV	MLR	5	Center-out right hand movements in horizontal 3D space.	Pearson’s r: x velocity:0.19 y velocity:0.38 z velocity:0.32
Kim et al. (2014a)	ME	Hand	EV	MLR, KRR^①^	4	Subjects were instructed to move their right arm continuously and along the infinity shape trajectory (∞) symbol and‘˄’ symbol when viewed from y-z axes and x-y axes	NRMSE: KRR:0.22 (2,400 samples) MLR:0.28 (2,400 samples)
Korik et al. (2015)	ME,MI	Hand	EV, BP	MLR, NN^②^	1	Repeated movement of right dominant hand between a home position and one of five target positions.	Pearson’s r: MLR:0.43 (best case) NN:0.73 (best case)
Korik et al. (2018)	ME,MI	Hand	EV, BP	MLR	12	Executed or imagined arm movements from the home position to target(four targets).	Pearson’s r: ME: BTS^③^:0.4 PTS^④^:0.15 MI: BTS:0.2 PTS:0
Sosnik and Zur (2020)	ME,MI	Hand, elbow, and shoulder	EV	MLR	7	Executed or imagined arm movements from the home position to four targets.	Pearson’s r: ME: Hand:0.24 ~ 0.49 Elbow:0.41 ~ 0.48 Shoulder:0.18 ~ 0.40 MI: Hand:0.09 ~ 0.23 Elbow:0.20 ~ 0.27 Shoulder:0.11 ~ 0.18
Sosnik and Zheng (2021)	ME,MI	Hand, elbow, and shoulder	BP	MLR	9	Executed or imagined arm movements from the home position to four targets.	Pearson’s r: ME:0.36 ± 0.13 MI:0.18 ± 0.11
Korik et al. (2016)	ME	Hand	EV, BP	MLR	3	Perform 15 hand movements between the home position and one of the six targets.	Pearson’s r: 0.45
Heger et al. (2012)	ME	Hand	FBCSP	MLR	5	Fill water into a glass.	Pearson’s r: velocity: x: 0.41 y: 0.36 z: 0.48 speed: 0.17
Kim et al. (2014b)	ME,MI	Arm	EV	MLR, KRR	10	Execute a motor trajectory, observe the trajectory performed by a volunteer’s hand and imagine the motor command for this trajectory, and to observe a trajectory performed by a robotic arm and imagine the movement.	-
Ofner and Müller-Putz (2012)	ME	Arm	EV	MLR	5	Perform natural, round, and in speed varying arm movements.	Pearson’s r: position: x: 0.70 ± 0.12; y: 0.78 ± 0.09 z: 0.62 ± 0.14 velocity: x: 0.70 ± 0.13 y: 0.77 ± 0.11 z: 0.62 ± 0.15
Pancholi et al. (2022)	ME	Hand	EV	WPD^⑤^, CNN^⑥^, LSTM^⑦^	12	Reach and grasp the object and lift it stably for a couple of seconds.	Pearson’s r: position: x: 0.86 y: 0.89 z:0.82
Jeong et al. (2020)	ME,MI	Arm	EV	CNN-BiLSTM	15	Perform and image center-out arm reaching in six directions.	Pearson’s r: ME:0.4712 MI: 0.4575 NRMSE: ME: 0.1780 MI: 0.1685
Shakibaee et al. (2019)	ME,MI	Knee	EV	NARX^⑧^ neural network	10	Extend and flex their right knee slowly at a constant speed. Imagine the movement of folding and unfolding the knee	MSE error: ME: 5.81E-07 MI: 2.36E-07

①Kernel ridge regression, ②Neural network, ③Band power time-series, ④potential time-series, ⑤Wavelet packet decomposition, ⑥Convolutional neural network, ⑦Long short-term memory, ⑧Nonlinear autoregressive exogenous.

### 3.2. EEG signal pre-processing

The 3D trajectory reconstruction task uses the same pre-processing method as the 2D trajectory reconstruction task, as detailed in Section 2.1.

#### 3.2.1. Filtering

Some studies have shown that low-frequency EEG signals carry information about limb movement (Korik et al., 2015, 2018). Therefore, low-pass filters or band-pass filters are used to pre-process EEG. In this work, the 3D motion information of the hand and arm is preserved by using the 0–1 Hz low-pass filter. In Presacco et al. (2011), the lower limb motion information is preserved by a 0.1–2 Hz band-pass filter. In Ofner and Müller-Putz (2012), the velocity and position of arm motion are decoded by low-pass filter and band-pass filter, respectively. In addition, a lot of work uses multi-band filtering to extract the power characteristics of EEG signals. In Korik et al. (2015), FFT is used to calculate the power of 0–4 Hz (delta), 4–8 Hz (theta), 8–12 Hz (mu), 12–18 Hz (low beta), 18–30 Hz (high beta), 30–40 Hz (low gamma), 60–84 Hz, 84–100 Hz and 100–150 Hz (high gamma) band, and put it into neural network decoding. In Korik et al. (2018), the frequency bands of delta (0.5–2 Hz), theta, mu, low beta, high beta (18–28 Hz), and gamma (28–40 Hz) are filtered, and the results are directly input to the PTS model. The band power of the filtered EEG signal is calculated and input into the BTS model. In order to discuss the frequency band which makes the greatest contribution to trajectory reconstruction, the lower delta band, including slow cortical potential (SCPs) (cutoff frequency is 1 Hz), higher delta band (1–4 Hz), theta band, alpha band (8–12 Hz) and lower beta band (12–15 Hz) are filtered. The multi-class filter library common space mode (FBCSP) algorithm is applied to the prediction of continuous output in Heger et al. (2012). A set of 4 Hz broadband pass filters are applied to the range from 1 to 28 Hz (1–4 Hz, 4–8 Hz, 8–12 Hz, 12–16 Hz, 16–20 Hz, 20–24 Hz and 24–28 Hz).

For the limb motion data, because the power range in the low-frequency band accounts for the vast majority of the total power of the signal, low-pass filtering is mainly used to process the limb motion data.

### 3.3. Decoding methods

#### 3.3.1. MLR

MLR is the most commonly used decoding method in 3D trajectory reconstruction. For a detailed introduction to MLR, see Section 2.4.2. Bradberry et al. (2010) first proposed to use MLR decoding EEG signals to reconstruct hand trajectories in Bradberry et al. (2010), which is called the PTS model method (Korik et al., 2018). Since then, it has been widely used to reconstruct various limb trajectories, such as lower extremities, hands, shoulders, elbows and arms. Korik et al. (2015) modified the PTS model, calculated the multi-band power density of the initial EEG signal, and proposed the BTS model (Korik et al., 2018).

#### 3.3.2. Kernel ridge regression

EEG limb trajectory reconstruction tasks are mostly limited to simple tracks, such as center-out tasks and point-to-point movement. However, in daily life, patients often have to do more complex movements. In KRR, the input data is mapped to the kernel feature space by mapping ∅. We used the kernel technique based on the Gaussian kernel function and defined variables:

(7) $$k\left(x\left(i\right),x\left(j\right)\right)=e^{-{\left(x\left(i\right)-x\left(j\right)\right)}^{2}/\sigma }$$

(8) $$p\left(t\right)=\{\begin{array}{c} x\left(t\right)-x\left(t-1\right)\\ y\left(t\right)-y\left(t-1\right)\\ z\left(t\right)-z\left(t-1\right) \end{array}$$

(9) $$s\left(t\right)={\left|\overset{C}{\underset{c=1}{\sum }}\overset{L}{\underset{k=0}{\sum }}S_{n}\left[c-k\right]\right|}^{T}$$

(10) y=[p(t=1),..,v(t=T)]

(11) X=[s(t=1),..,s(t=T)]

where σ is the width of the Gaussian kernel function, *p* is the *w* position of time *t* on the *x*, *y*, and *z* axes, *s* is the normalized EEG data, *L* is the number of time lags, and *C* is the number of electrodes used for analysis.

Kim et al. (2014a,b) used the non-linear method Kernel Ridge Regression (KRR) to decode complex motion trajectories. KRR uses the kernel method to map input data to a kernel feature space, which is widely used in the field of robot motion control (Ofner and Müller-Putz, 2012). In Kim et al. (2014a), Kim used the Gaussian kernel function to define kernel functions with multi-channel EEG time series as input, motion speed and trajectory position as output. The results show that KRR achieves better decoding accuracy than the linear method in reconstructing complex motion which is highly related to real scene, and KRR can also produce better results when the number of training samples is small, and the computational cost is significantly reduced.

#### 3.3.3. Artificial neural network

In the past few years, deep learning, a sub-field of machine learning, has achieved breakthrough in complex and high-dimensional data, such as image classification (Zhang et al., 2019) and emotion recognition (Li et al., 2019). Compared with linear decoding methods, deep learning models infuse non-linearity by adding nonlinear activation functions in the hidden and output nodes. So that they can access very descriptive (nonlinear) features that define the underlying relationships quite well (Irimia et al., 2018). Neural networks have been popular in MTP tasks, which could be used to reconstruct complex motion trajectories.

Korik et al. (2015) used a neural network to decode the 3D motion trajectory of the hand (Korik et al., 2015). It is considered that the time evolution of the spectrum power value contains more valuable information than the original EEG potential, so the spectrum power value is used as the input. The paper also sets up many control groups, including potential-based NN, spectrum power-based MIMO and MISO architecture NN, potential-based MLR, and spectrum power-based MLR. Finally, the proposed neural network model based on spectrum power value achieves the highest decoding accuracy of approximately 0.7.

Shakibaee et al. (2019) used NARX neural network to decode knee angle trajectory. The NARX is a nonlinear dynamic neural network model, which combines Autoregressive Exogenous (ARX), polynomial nonlinear function and Classical Gram Schmidt (CGS) orthogonalization method. NARX model is used in modeling the time series, meaning that the current value of a time series (output) can be predicted by the previous values of the same times series (output at previous moments), along with the current and previous values of the exogenous input. It has nonlinearity, dynamicity, tractability, and simplicity trait, which makes it an appropriate model for online MTP-BCI application. The NARX recursive neural network has two structures: parallel and series. In this research, Shakibaee et al. (2019) used series structure to get a better result of 5.81E-07 MSE error in ME and 2.36E-07 MSE error in MI.

The hybrid deep learning model using the CNN and the LSTM performed fairly well in MTP-BCI field (Irimia et al., 2018; Pancholi et al., 2022) for extracting spatio-temporal quality features. Jeong et al.(Pancholi et al., 2022) proposed MDCBN (Multi-Directional CNN-BiLSTM Network) framework to decode hand velocity for six directions in 3D space and control a robotic arm based on the result. Conventional hybrid deep learning frameworks (Tabar and Halici, 2016; Li et al., 2018; Wang et al., 2018; Shakibaee et al., 2019) usually trained CNN to extract spatial features of brain activities and trained LSTM for temporal information. In contrast, MDCBN uses a CNN architecture to train the multi-direction information per axis as pre-training and the BiLSTM network for training the relationships in the 3D space (x-, y-, and z-axes). This research adopts a subject-dependent BCI and got a decoding result of 0.4712 in ME and 0.4575 in MI. Pancholi et al. proposed a CNN-LSTM framework based on wavelet packet decomposition (WPD) for hand kinematics prediction (Irimia et al., 2018). WPD could decompose he EEG signal into sub-bands with increasing resolution toward the lower frequency band (Zhang et al., 2017; Khalil et al., 2019), which is considered to carry detailed limb kinematic information. Despite the high computational cost and a large amount of training data, this model got an extremely high accuracy of 0.86, 0.89, and 0.82 in the *x*-, *y*-, and *z*-axes, respectively.

## 4. Discussion

In the past decade, many achievements have been made in EEG-based limb trajectory reconstruction, and it is possible to use EEG to control the continuous movement of external prosthetic devices, which brings good news to patients with spinal cord injuries and other people with severe mobility. However, there are still many problems to be further explored, such a how to reconstruct complex motion trajectories and which frequency bands carry motion information and artifacts influence. In order to further promote the practical application of MTP, we believe that these important problems should be solved in future work. In this section, we briefly summarize the main findings of this review and introduce these research directions and current developments. It also illustrates the basic challenges and some potential ideas.

### 4.1. Summary of major findings

In this section, we summarize the data processing flow and common algorithms in the task of limb trajectory reconstruction, to provide a reference for readers when designing algorithms. In the healthy subjects reconstruction process, the MLR and Kernel Ridge Regression models are fast in operation and efficient in decoding. The artificial neural network model for reconstructing simple motion trajectories has high decoding accuracy.

Data quality, feature selection, and decoding methods may have a great impact on the accuracy of reconstruction results. In order to obtain high-quality EEG signals, data pre-processing is an indispensable step. Filtering and ICA are the two most common pre-processing methods. Since it is generally believed that limb motion information exists in low-frequency EEG signals, the low-pass filter of 2 Hz are often used in pre-processing. ICA is used to eliminate the influence of ophthalmogram, EMG, and other signals. Manual removal is a common method.

The purpose of feature extraction is to achieve better results in the following decoding process. The amplitude of EEG signal is the most commonly used feature, which can be decoded after simple standardized processing. Some studies have used signal processing methods, such as DWT (Robinson et al., 2013; Robinson Neethu et al., 2014; Robinson et al., 2015) and DSP, CSP (Bradberry et al., 2010; Lv et al., 2010), for processing amplitude signals. Band power is also a common feature in the frequency domain. In addition, phase features have also been used (Sun et al., 2017).

Choosing the appropriate decoding method is the key to obtaining good reconstruction results. In the existing work, MLR is the most popular decoding method. MLR has the advantages of simple principles and strong applicability. MLR can decode the amplitude feature, band power and phase feature reading and obtain satisfactory results. Kalman filter is used to reconstruct 2D motion trajectory, which can optimize the model and estimate the current state of the system through new observations. PLS is usually used to solve the problem of multiple collinearities caused by large channel correlation (Ofner and Müller-Putz, 2014; Mondini et al., 2020). KRR achieves good decoding accuracy when reconstructing complex motion that closely resembles real-life scenarios, and KRR can also produce better results when the number of training samples is small, and significantly reduce the computational cost (Kim et al., 2014a). In addition to KRR, neural networks are also used to reconstruct complex motion trajectories and produce fairly high decoding accuracy when using band power as input.

### 4.2. Current issues and future considerations

#### 4.2.1. Artifact influence

Using ICA to remove EOG, and EMG components is a very common EEG pre-processing method. However, many studies have found that using ICA to remove artifacts will reduce the correlation coefficient of the reconstructed trajectory. Given this phenomenon, the popular view is that the motion performed may have some effect on the signal in the low-frequency band, that is, the actual physical motion distortion on the EEG electrode. The artifacts generated during the execution of the motion exist in many independent sub-spaces decomposed by ICA, which is difficult to remove. The use of over-sensitive ICA will lead to the destruction of EEG signals, which results in a reduction of decoding accuracyy. Concerning the effect of eye movement, Kim et al. (2014b) show that some ICA components show a strong correlation with EOG signals, which are being used by decoders when EOG-related activities are left in the EEG.

In order to reduce the impact of artifact removal on reconstruction accuracy, some work uses nonlinear decoding methods, such as KRR, and neural networks. The results show that the decoding performance of nonlinear methods is less affected by artifact removal. Because the execution motion may have some influence on the low-band signal, and most of the reconstruction work uses the low-frequency EEG signal as input to the decoder, we can try to use the signal of other frequency bands, using the band power density, phase and other characteristics as the input to weaken the influence of the low-frequency signal. Although the use of other features and other decoding methods can reduce the impact of artifacts, the mechanism of the impact of artifacts on EEG is still unknown, which still remains an open question.

#### 4.2.2. Motion variability and complex motion

Most of the research in the field of limb trajectory reconstruction focuses on decoding some low-speed, simple motion trajectories, such as center outward movement. However, in daily life, we usually need to perform some tasks with complex trajectories. Conventional linear methods are not effective in decoding such tasks. Some studies have shown that there is a negative correlation between motion variability and trajectory reconstruction accuracy in the process of limb trajectory reconstruction. Bradberry et al. (2010) offer two possible explanations: from the point of view of machine learning, the reason that higher motion variability will lead to lower decoding accuracy is that the EEG-kinematics samples of complex motion have low similarity, while simpler movements lead to an increase in the number of similar training samples, and the training effect of the decoder is better. From the point of view of neuroscience, the subjects have different abilities to perform tasks without practice, so the intensity of the prior neural representation of the movement required is different. These different strengths may be directly related to the accuracy of the extracted representation.

We can use sensorimotor integration of multi-dimensional sensory stimulation as an instruction to execute or imagine complex motion trajectories (Li et al., 2022). Unlike the simple sensory cue instructions used in most previous work, sensorimotor integration combines multiple sensory commands such as vision and hearing, Mazurek et al. (2019) can deliver information to the subjects in a time series. They have found ERPs that differentiate the instruction used and the action performed in neural activity near motor cortex and posterior parietal cortex in the left hemisphere. Delivering complex sensor stimulation to be used as instructions for performing detailed actions, we can accurately describe the changes in kinematics trajectory and EEG signal, which may be helpful to improve the reconstruction accuracy.

For complex movements (e.g., stroke gait, gait in the elderly), the direct reconstruction may lead to a decrease in accuracy due to the irregular trajectory of the extremities. We can use biomechanical models of the lower limb to capture the intrinsic joint angles performed, and indirectly reconstruct the end trajectory by reconstructing the angle, torque, and other parameters, which has been adopted in previous studies (Presacco et al., 2011, 2012; Mazurek et al., 2019; Mercado et al., 2021).

In order to improve the ability to decode complex motion trajectories, some nonlinear methods, such as KRR (Kim et al., 2014a,b), and NN (Korik et al., 2015), can significantly improve the decoding accuracy, but the computational overhead is also increased. In addition, why the nonlinear decoding method can produce better accuracy is also a problem to be studied. One explanation put forward by Kim et al. (2014b) is that we cannot represent all the trajectories needed under realistic conditions in the linear subspace of EEG activities. The kernel method has been proven to be very effective for motor control tasks in robots, which shows that motor control with complex trajectories can be better modeled using nonlinear models. Additionally, we cannot completely rule out the possibility that the nonlinear decoder uses non-neural signals that cannot be accessed by the linear decoder in the EEG data.

The realization of real-time and accurate decoding of complex trajectories is an important step in the practical application of MTP-BCI, which requires us to constantly optimize the performance and delay of decoding methods. Our understanding of the key characteristics of different trajectories and which neural signals are used by decoders is still very limited, which is a very important issue, which involves the mechanism of motion control and can provide guidance for us to design decoders. Solving this problem requires the joint efforts of more researchers.

#### 4.2.3. Reconstruction in patients movements

Stroke has brought a heavy burden to patients, families, and society, and the recovery after stroke is usually incomplete. Improving the recovery and long-term outcomes after stroke has become an important challenge for clinical and BCI applications. Trajectory reconstruction of stroke is great significance for patients’ rehabilitation and assisting patients’ movement.

Di Marco et al. (2021) recruited stroke survivors and a sex-and age-matched control group, undergo a single training session with an active exoskeleton for gait rehabilitation, and recorded EEG, MEG, and gait characteristics before and after training. By analyzing the EEG signals during gait, they found the negative deviation of low EEG frequency (0.1–3 Hz) before the start of exercise, that is, the motor-related cortical potential (MRCP). This kind of biomarker can be used as a reliable predictor of lower limb movement, which can help to more accurately divide the EEG signals during trajectory reconstruction. Furthermore, the negative amplitude of MRCP is related to participants’ level of participation in performing exercise tasks, which can help evaluate the quality of EEG signals during trajectory reconstruction.

Alpha and beta rhythms are cortical rhythms that are mainly involved in exercise planning and control, and the movement disorder of stroke survivors is the main factor leading to disability. In Di Marco et al. (2021)'s experiment, the frequency band power density of the alpha and beta bands of the control subjects increased after training. This result inspired us to extract features from higher frequency band (> 8 Hz) signals for trajectory reconstruction or increase the proportion of cortical activity signals in sensory-motor areas during signal preprocessing (Section 2.2) and feature extraction (Section 2.3). Moreover, because the gait of stroke is usually complicated, the nonlinear decoding method in Section 3.3, such as KRR and NN, can be selected as the decoding method.

In addition, research shows that the effective connectivity between the stroked motor area and other areas degraded in patients when compared to healthy controls, and after rehabilitation training, the connectivity between the non-stroke motor area and other areas, especially the frontal lobe and parietal-occipital lobe, is enhanced (Sadiq et al., 2019). This discovery can not only guide us to choose suitable electrodes for trajectory reconstruction, but also provide a reliable biomarker for the rehabilitation effect.

As far as the author knows, there is no work to reconstruct the trajectory directly by using the EEG signals of stroke or other neurological populations. These clinical populations are the main users of MTP-BCI applications, and this work needs the supplement of BCI researchers.

#### 4.2.4. Frequency band analysis

Determining the frequency band of motion information in EEG signals is an important issue in MTP-BCI research. Existing studies have drawn different conclusions on this issue, which mainly depends on the selected features and decoding methods. The commonly used features can be divided into two categories, potential time series (PTS) and band power time series (BTS). If PTS model is selected, MLR decoding method can usually achieve the highest decoding accuracy in delta band. Many studies based on the Bradberry model (Bradberry et al., 2010) confirm this, showing the special status of the delta band. However, this conflicts with a large body of literature on classical SMR-BCI, which reports the highest accuracy using the power values of mu and beta bands. However, the PTS model can not only be decoded in the delta band. Korik et al. (2015) use the PTS model based on a nonlinear feed-forward neural network to achieve high decoding accuracy in most sub-gamma bands (< 40 Hz), which is consistent with the research in the field of SMR. If we choose the BTS model for executing and imagining motion, many studies have shown that it can achieve high decoding accuracy in the low gamma band of mu and beta (Korik et al., 2015, 2018), which is consistent with the research results in the SMR field, and the accuracy is significantly higher than that of the PTS model.

Using the linear regression model to adjust the two-time signals requires that the two signals span the same frequency range. Therefore, it has been proposed that the good decoding performance in the delta band is because the experimental paradigm usually involves periodic arm motion at low speed (0.5–2 Hz), rather than carrying motion information. The result of shuffling tests (Sosnik and Zur, 2020) negates this claim, but the decoding performance of the delta band is indeed highly modulated by motion planning and generation. For the potential signal in the intermediate band (mu, beta), because of its low SNR and the reconstruction process depending on a short time window, it is difficult to reconstruct the trajectory (Sosnik and Zur, 2020). However, when using the BTS model, the decoding accuracy in mu and beta bands is significantly higher than that in delta bands. At present, the correlation of trajectory reconstruction obtained by BTS model is significantly higher than that of PTS model, so BTS model has become potential.

The EEG signal is nonlinear and non-Gaussian, so the mathematical relationship between EEG and limb motion will be complex. We still do not know why the BTS model has better performance, and the relationship between frequency band and motion is still an open problem worth exploring. We have achieved excellent decoding performance in these frequency bands, so we have reason to believe that the research in this area is promising.

#### 4.2.5. Generalization performance

Improving the applicability of decoding methods to different users and the generalization performance in different environments is an important topic in MTP-BCI. Subjects need to receive real-time feedback from external devices when performing motion imagination or motion execution tasks to obtain the perception of the adaptability of the BCI system, and dynamically adjust the attention and control mode (for example, the speed of movement). The control effect often varies from person to person and requires a certain amount of training. In order to improve the generalization performance of the decoder, we expect to modify the regression weight through the EEG activity (Bradberry et al., 2010). However, for other nonlinear methods, there are still gaps in this part of the research, looking forward to the exploration of more researchers.

#### 4.2.6. Kinesthetic memory

Repeating the same action may lead to strong kinesthetic memory, in which subjects use joint muscle memory for motor imagination. Kinesthetic memory may lead to the evolution of separate and different neural patterns of different joint trajectories, which allows them to be reconstructed. This explanation is consistent with our experience and has been supported by some research results (Sosnik and Zur, 2020). The accuracy of trajectory reconstruction enhanced by muscle memory is a very attractive conjecture, but its effectiveness and induction methods for people with motor disorders still need to be further studied.

#### 4.2.7. Pathological damage

Most of the MTP-BCI studies were conducted on healthy subjects, but it is not easy to transfer the results of healthy subjects to patients with dyskinesia (due to stroke, neurological disease, or brain trauma), which is the target group of MTP-BCI. Because there is no guarantee that the response of the normal BCI paradigm to motor intentions in healthy subjects’ brain activity will normally behave the same in patients with brain damage, it is not clear how best MTP-BCI uses the user’s EEG signal (Sosnik and Zheng, 2021). However, many studies (Gramfort et al., 2013; Cantillo-Negrete et al., 2018; Shu et al., 2018) have shown that the results obtained from healthy subjects can be used to shorten the calibration phase of patients with motor impairment, and similar factors contribute to the decoding of motor imagination. Future work should test the applicability of the results to patients with exercise impairment and modify the model pertinently.

## 5. Conclusion

MTP-BCI is ideal for controlling the continuous motion of the external prosthesis. This study provides a comprehensive review of it, focusing on the process of trajectory reconstruction. The latest development and limitations of data pre-processing, feature extraction, and decoding methods are introduced, which provides a feasible reference for future research. In addition, we discuss the main findings of this study. Finally, we list the open problems and trends that need to be further studied from the aspects of artifact influence, complex trajectory reconstruction, frequency band range, generalization performance, kinesthetic memory, and pathological damage. Overall, we believe that MTP-based BCI has strong application potential for disability assistance and rehabilitation for people with disabled individuals, and there is still much room for improvement in decoding complex tasks.

## Author contributions

DZ and PW designed the study. XC and PW wrote the manuscript. PW, YZ, and PG collected the relevant literature. XC, MY, and YZ prepared the figures. WS and DZ reviewed and edited the manuscript. All authors contributed to the article and approved the submitted version.

## Funding

This work was supported in part by the National Natural Science Foundation of China (NSFC) under Grant 62136004, Grant 61876082, and Grant 61732006; in part by the National Key Research and Development Program of China under Grant 2018YFC2001600 and Grant 2018YFC2001602; and in part by the Research Fund for International Young Scientists (NSFC Grant No. 62050410348).

## Conflict of interest

The authors declare that the research was conducted in the absence of any commercial or financial relationships that could be construed as a potential conflict of interest.

## Publisher’s note

All claims expressed in this article are solely those of the authors and do not necessarily represent those of their affiliated organizations, or those of the publisher, the editors and the reviewers. Any product that may be evaluated in this article, or claim that may be made by its manufacturer, is not guaranteed or endorsed by the publisher.
